# Perceived AI interactivity and motivated learning behaviors in AI-Supported EFL learning in higher education: the mediating roles of flow and self-efficacy

**DOI:** 10.3389/fpsyg.2026.1871610

**Published:** 2026-08-12

**Authors:** Min Guo, Jiazhu Li, Yang Yang, Niu Sun

**Affiliations:** 1 School of Foreign Languages, Xinyang Normal University, Xinyang, China; 2 School of Computer and Information Technology, Xinyang Normal University, Xinyang, China

**Keywords:** AI-supported EFL learning, flow experience, motivated learning behaviors, perceived AI interactivity, self-efficacy

## Abstract

This study examined the relationship between perceived AI interactivity (PAII) and motivated learning behaviors (MLBs) among English as a Foreign Language learners in China, with particular attention to the mediating roles of flow experience and self-efficacy. Data from 290 valid responses collected through online questionnaires were analyzed using descriptive statistics, correlation analysis, and partial least squares structural equation modeling (PLS-SEM). The results showed that PAII was not directly associated with MLBs, but exerted significant indirect effects through self-efficacy and through the sequential pathway involving flow experience and self-efficacy. By contrast, the indirect effect through flow experience alone was not significant. Specifically, higher levels of PAII were associated with stronger flow experience, which in turn was associated with higher self-efficacy and subsequently with stronger MLBs. These findings highlight the importance of flow experience and self-efficacy in explaining how perceived AI interactivity is associated with MLBs. The study also offers practical implications for the design of AI-supported learning environments and for teaching practices aimed at fostering learners' flow experience and self-efficacy.

## 1 Introduction

Artificial intelligence (AI) tools, such as conversational agents, intelligent tutoring systems, and automated writing feedback tools, are rapidly transforming language education (Wiboolyasarin et al., 2025). By providing real-time and adaptive feedback, these tools extend learner support beyond traditional instruction (Mohebbi, 2025). However, existing research suggests that the educational value of AI tools cannot be understood from their availability alone (Chen et al., 2024). It also depends on how learners perceive their interaction with these tools in learning contexts (Wang et al., 2025). As a result, increasing attention has been given to how learners perceive their interaction with AI tools.

Perceived AI interactivity (PAII) has been proposed as a key construct for describing learners' evaluations of their interaction with AI tools, including responsiveness, controllability, engagement, and personalization (Wang et al., 2025). Previous studies have shown that learners' perceptions of their interaction with AI tools are positively related to their participation and engagement in learning (Wang et al., 2023; Hidayat-ur-Rehman, 2024). In the present study, learners' behavioral engagement is examined through motivated learning behaviors (MLBs), which reflect sustained behavioral engagement in learning activities. Prior research has also shown that PAII is positively associated with EFL learners' MLBs (Guo and Li, 2025). However, the underlying mechanisms through which PAII may be associated with learners' MLBs remain insufficiently explained.

One possible explanation for this relationship may lie in learners' personal psychological processes in AI-supported learning. In the present study, these processes are represented by two complementary psychological aspects: flow experience and self-efficacy. These two constructs were selected because they capture different but closely connected dimensions of learners' psychological responses to AI-supported EFL learning. Flow experience reflects learners' immediate experiential states during task engagement, such as deep absorption, enjoyment, and a sense of control (Csikszentmihalyi et al., 2014). Accordingly, flow experience is particularly relevant to understanding learners' immediate responses to AI-supported interaction, as interaction features such as responsiveness and engagement may help learners become more immersed in learning and more likely to experience flow (Zhou et al., 2025). Self-efficacy, by contrast, reflects learners' beliefs in their ability to accomplish learning tasks successfully (Bandura, 1997). In AI-supported learning, timely feedback and personalized support may strengthen learners' efficacy beliefs by providing mastery experiences and a stronger sense of control (Qiu, 2025). Self-efficacy is closely associated with sustained behavioral engagement and is therefore relevant to explaining MLBs (Schunk and DiBenedetto, 2020). Together, flow experience and self-efficacy represent two complementary psychological mechanisms through which PAII may be associated with MLBs: flow experience during AI-supported learning and learners' efficacy beliefs in completing learning tasks.

Social Cognitive Theory (SCT) provides an appropriate theoretical framework for understanding these mechanisms because it explains how learning environments, personal psychological processes, and motivated behaviors are connected (Bandura, 1986). From an SCT perspective, learners' behavior is not shaped by environmental conditions alone, but also by how learners perceive, interpret, and respond to those conditions. In the present study, PAII represents learners' perceptions of AI interactivity within AI-supported learning environments, while flow experience and self-efficacy represent two personal psychological processes through which such perceptions may be connected with MLBs. This theoretical logic suggests that PAII may not directly translate into MLBs; rather, its association with MLBs may depend on whether learners experience immersive learning states and develop stronger efficacy beliefs. Prior research further suggests that positive learning experiences marked by deep absorption, enjoyment, and a sense of control may support stronger self-efficacy beliefs (Jia et al., 2024; Su et al., 2024). Therefore, SCT offers a suitable framework for explaining why flow experience and self-efficacy may mediate the relationship between PAII and MLBs, with a possible sequential relationship between the two.

Based on SCT, the present study aims to examine the relationship between PAII and English as foreign language (EFL) learners' MLBs by focusing on the mediating roles of flow experience and self-efficacy among Chinese EFL learners. Specifically, it addresses the following research question: How do flow experience and self-efficacy mediate the relationship between perceived AI interactivity and motivated learning behaviors among Chinese EFL learners? This study contributes to the literature by offering a clearer explanation of how PAII is associated with MLBs through personal psychological processes, particularly flow experience and self-efficacy. The findings also have practical implications for teachers and AI designers seeking to better support learners' MLBs. In particular, AI-supported learning experiences should be designed in ways that foster learners' flow experience and strengthen their self-efficacy.

## 2 Literature review

### 2.1 Perceived AI interactivity in EFL learning contexts

Perceived AI interactivity (PAII) has emerged as an important construct in research on AI-supported learning. Critically, PAII refers not to the technical functions of AI tools, but rather to learners' subjective perceptions of AI interactivity (Wang et al., 2025). This distinction is important because the educational value of AI-supported learning depends not only on what AI tools are designed to do, but also on how learners perceive and experience interacting with them. Wang et al. (2025) conceptualized PAII as a multidimensional construct comprising four dimensions: (1) responsiveness, referring to the perceived timeliness and relevance of AI feedback; (2) controllability, concerning learners' perceived ability to guide or regulate interaction; (3) engagement, reflecting the extent of learners' involvement during interaction with AI tools; and (4) personalization, referring to the degree to which AI tools adapt to learners' needs. Together, these dimensions capture learners' perceptions of AI interactivity in learning contexts.

PAII is especially relevant in EFL learning, where AI-supported learning often relies heavily on interaction (Wiboolyasarin et al., 2025). Learners use AI tools not only to access information, but also to ask questions, receive feedback, revise output, and sustain learning activity across tasks and contexts (Mohebbi, 2025). Under such conditions, the perceived quality of AI interactivity is likely to shape learners' learning experiences and their behavioral engagement in learning (Chen et al., 2024; Wang et al., 2023; Hidayat-ur-Rehman, 2024). However, the relationship between PAII and learning engagement may depend on intervening psychological processes rather than reflecting a simple direct association. Recent research on AI-generated feedback suggests that timely and interactive feedback does not necessarily suggest meaningful student engagement, as learners' feedback literacy and evaluative judgment remain important for engaging with AI feedback effectively (Zhan et al., 2025). Similarly, studies on AI dialogue systems have warned that over-reliance on AI may reduce learners' critical evaluation of AI-generated responses and weaken deeper cognitive engagement (Zhai et al., 2024). Therefore, PAII may not directly translate into MLBs unless learners' perceptions of AI interactivity are connected with relevant psychological processes. Although PAII has been associated with MLBs, the psychological mechanisms underlying this association remain insufficiently explained.

### 2.2 Motivated learning behaviors

Motivated learning behaviors (MLBs) are defined as learners' observable effort, persistence, and proactive engagement in goal-directed learning activities (Gagné and Deci, 2005). MLBs are especially important in language learning, where progress often depends on sustained participation, continued effort, and active engagement with learning tasks and resources (Reinders and White, 2011). In AI-supported learning environments, such behavioral involvement may be shaped by how they perceive and experience interaction with AI tools (Wang et al., 2023). Recent studies have shown that learners' experiences in AI-supported learning are related to their participation and engagement in learning activities (Hidayat-ur-Rehman, 2024). More directly, Guo and Li (2025) found that PAII was positively associated with EFL learners' MLBs, with higher levels of PAII linked to stronger MLBs. Relatedly, Shao and Chen (2021) reported that learners' positive perceptions of interactivity were associated with stronger sustained engagement in learning. Taken together, these findings suggest that learners who perceive AI-supported interaction more positively are more likely to demonstrate stronger MLBs. Therefore, the following hypothesis is proposed:

**H1:** PAII is positively associated with EFL learners' MLBs.

### 2.3 Flow experience

Flow experience refers to a state of enjoyment, deep absorption, and a sense of control during task engagement (Csikszentmihalyi et al., 2014; Schmidt et al., 2014). It typically arises when individuals perceive a balance between task demands and their own skills (Rodríguez-Ardura and Meseguer-Artola, 2017). In AI-supported learning, such a state may be more likely to occur when learners perceive interaction with AI tools as responsive, manageable, and engaging. These interaction features may help learners become more immersed in learning and more likely to experience flow (Zhou et al., 2025). In turn, flow experience may be associated with MLBs. When learners experience deep absorption, enjoyment, and a sense of control during learning, they may be more likely to sustain effort, persist in tasks, and remain proactively engaged in learning activities. However, this positive association should not be understood as uniform across learning contexts. Flow experience reflects learners' positive state during task engagement, whereas MLBs involve sustained behavioral engagement in learning activities (Gagné and Deci, 2005; Li et al., 2021). This conceptual distinction suggests that flow experience may explain part of the psychological pathway through which PAII is associated with MLBs. Prior research has shown that flow is positively associated with learning engagement in AI-supported tasks (Yang et al., 2025) and may help explain the relationship between learners' psychological conditions and engagement in learning (Gao et al., 2025). In sum, these findings suggest that flow experience provides a plausible psychological pathway through which PAII may be associated with learners' MLBs:

**H2:** Flow experience mediates the relationship between PAII and MLBs.

### 2.4 Self-efficacy

Self-efficacy reflects learners' beliefs about their ability to perform learning tasks successfully (Bandura, 1997). It is widely recognized as a central factor associated with learners' motivated learning behaviors in academic settings (Margolis and McCabe, 2003; Gebauer et al., 2021) . Self-efficacy is relevant because learners often need to judge how effectively they can use AI tools to support their learning. Previous research suggests that when learners perceive AI-supported interaction as useful, controllable, timely, relevant, and personalized, they may feel more capable of managing learning tasks and develop a stronger sense of control, which can support self-efficacy (Du, 2025; Qiu, 2025). Nevertheless, the extent to which PAII contributes to self-efficacy may depend on learners' ability to interpret AI feedback and apply AI support to task completion. Given its close association with MLBs, self-efficacy may provide a plausible explanation for how PAII may be associated with learners' MLBs. Accordingly, the following hypothesis is proposed.

**H3:** Self-efficacy mediates the relationship between PAII and MLBs.

### 2.5 The mediating roles of flow experience and self-efficacy

Social Cognitive Theory (SCT) provides an appropriate framework for explaining the mediating roles of flow experience and self-efficacy in the relationship between PAII and MLBs (Bandura, 1986). Although frameworks such as Self-Determination Theory, the Technology Acceptance Model, and Expectancy-Value Theory can explain learners' motivation, technology acceptance, or achievement-related choices (Davis, 1989; Ryan and Deci, 2000; Eccles and Wigfield, 2002; Ryan and Deci, 2000), they are less directly aligned with the present study's focus on how PAII is associated with MLBs through personal psychological processes. SCT is therefore more suitable because it emphasizes the connections among perceived environmental conditions, personal psychological processes, and behavioral outcomes (Bandura, 1986).

From an SCT perspective, learners' behavior is not shaped by the learning environment alone, but also by how learners perceive, interpret, and respond to that environment. In this study, PAII represents learners' perceptions of AI interactivity within AI-supported learning environments, while flow experience and self-efficacy represent two personal psychological processes through which such perceptions may be connected with MLBs. This theoretical framework suggests that PAII may not be directly associated with MLBs; rather, its association with MLBs may depend on whether learners experience flow and develop stronger self-efficacy beliefs during AI-supported learning.

More specifically, flow experience and self-efficacy may be sequentially related. In the present study, flow experience is positioned before self-efficacy because PAII is assumed to first shape learners' immediate experiential state during AI-supported EFL learning. When learners perceive AI-supported interaction as responsive, controllable, engaging, and personalized, they may be more likely to experience concentration, enjoyment, perceived control, and smooth task engagement, which are central characteristics of flow experience (Csikszentmihalyi et al., 2014; Schmidt et al., 2014; Wang et al., 2025). Flow experience may then contribute to self-efficacy because learners may interpret fluent and manageable learning experiences as evidence that they are capable of completing EFL learning tasks successfully (Bandura, 1997; Jia et al., 2024; Su et al., 2024). Thus, flow experience is treated as an immediate experiential response to PAII, whereas self-efficacy is treated as a subsequent cognitive-motivational belief shaped by learners' interpretation of such experiences. In this sense, flow experience functions as an immediate experiential mechanism through which PAII may support the development of self-efficacy, which subsequently contributes to MLBs. Accordingly, the following hypothesis is proposed:

**H4:** Flow experience and self-efficacy sequentially mediate the relationship between PAII and MLBs.

## 3 Methodology

A cross-sectional quantitative research design was employed to test the proposed hypotheses. This study examined the relationship between PAII and Chinese EFL learners' MLBs, focusing on the mediating roles of flow experience and self-efficacy.

### 3.1 Subjects

Participants were 290 Chinese EFL learners recruited through convenience sampling from a Chinese normal university. Participants were included if they were enrolled university students learning English as a foreign language and agreed to participate voluntarily. The participants' demographic characteristics are summarized below (see Table 1). The sample included 228 female students and 62 male students. In terms of academic major, 195 participants were English majors and 95 were non-English majors. The sample also represented multiple academic levels, including freshmen (*n* = 112), sophomores (*n* = 72), juniors (*n* = 40), seniors (*n* = 48), and graduate students (*n* = 18).

**Table 1:** Demographic characteristics and AI-use information.

Variables	Category	*N*	%	Variables	Category	N	%
Gender	Male	62	21.4	Major	English major	195	67.2
	Female	228	78.6		Non-English major	95	32.8
Grade	Freshman	112	38.6	EP	CET−4	28	9.7
	Sophomore	72	24.8		CET−6	89	30.7
	Junior	40	13.8		TEM−4	6	2.1
	Senior	48	16.6		TEM−8	5	1.7
	Graduate students	18	6.2		None	162	55.9
AIUD	Never	1	0.3	AIUF	Never	3	1
	< 0.5 h/day	87	30		1–2 times/day	98	33.8
	0.5–1 h/day	129	44.5		3–4 times/day	113	39
	1–2 h/day	59	20.3		5–6 times/day	36	12.4
	> 2 h/day	14	4.8		≥7 times/day	40	13.8

*N* = 290. Percentages may not total 100% due to rounding. EP, English proficiency; AIUD, AI usage duration; AIUF, AI usage frequency; CET, college english test; TEM, test for english majors.

Participants reported varying levels of English proficiency, including CET-4 (*n* = 28), CET-6 (*n* = 89), TEM-4 (*n* = 6), TEM-8 (*n* = 5), and no formal English certification (*n* = 162). Participants also reported their AI-use experience, including the frequency and duration of AI use for academic learning purposes. Participants also reported using different types of AI tools for English learning, including AI chatbots such as ChatGPT, DeepSeek, and Kimi, as well as grammar checkers, translation tools, and automated writing feedback tools.

### 3.2 Research instrument

An online questionnaire was adopted to investigate subjects' PAII, flow experience, self-efficacy, and MLBs (see Appendix). Except for the demographic and AI usage items, all items were rated on a five-point Likert scale ranging from 1 (strongly disagree) to 5 (strongly agree). PAII was measured with a 10-item scale adapted from Wang et al. (2025), designed to assess learners' perceptions of the responsiveness, controllability, engagement, and personalization of AI tools in learning contexts. Flow experience was measured with a 5-item scale informed by flow theory (Csikszentmihalyi et al., 2014), capturing learners' absorption, enjoyment, and focused engagement during AI-supported learning. Self-efficacy was assessed using a 5-item scale grounded in Bandura, 1997 self-efficacy theory, focusing on learners' perceived capability to successfully carry out learning activities with AI support. MLBs were measured using a 4-item scale adapted from Wang and Wang (2022), assessing persistence, effort, and active engagement in learning.

The psychometric properties of the measures were then assessed. As shown in Table 2, all constructs demonstrated satisfactory internal consistency, with Cronbach's α and composite reliability values exceeding the recommended threshold of 0.70. Convergent validity was also supported, as all average variance extracted values were above 0.50. In addition, discriminant validity was assessed using the heterotrait-monotrait ratio (HTMT). As reported in Table 3, all HTMT values were below 0.85, supporting adequate discriminant validity among the four constructs. Moreover, all indicator outer loadings exceeded 0.70, and no problematic cross-loadings were observed, indicating acceptable indicator reliability.

**Table 2:** Reliability and convergent validity.

Dimensions	Cronbach's α	CR	AVE
PAII	0.909	0.923	0.524
FE	0.851	0.895	0.632
SE	0.868	0.905	0.655
MLBs	0.859	0.904	0.702

PAII, perceived AI interactivity; FE, flow experience; SE, self-efficacy; MLBs, motivated learning behaviors; CR, composite reliability; AVE, average variance extracted.

**Table 3:** Discriminant validity (HTMT).

Dimensions	PAII	FE	SE	MLBs
PAII	—	0.743	0.752	0.593
FE		—	0.715	0.625
SE			—	0.771
MLBs				—

PAII, perceived AI interactivity; FE, flow experience; SE, self-efficacy; MLBs, motivated learning behaviors; HTMT, heterotrait–monotrait ratio.

### 3.3 Data collection

Data were collected through *Wenjuanxing*, a widely used online survey platform in China, between November and December 2025. Participation was voluntary and anonymous, and informed consent was obtained before the questionnaire began. The questionnaire took approximately 10–15 min to complete. A total of 320 responses were initially collected through an online questionnaire, and 290 valid responses from students at a Chinese normal university were retained for analysis.

### 3.4 Data analysis

Data analysis was conducted using *SPSS 27.0* (IBM Corp., Armonk, NY, US) and *SmartPLS 4.0* (SmartPLS GmbH, B6nningstedt, Germany). SPSS was used to generate descriptive statistics and Pearson correlation coefficients for the four focal variables. The hypothesized model was tested using partial least squares structural equation modeling in *SmartPLS*, which is appropriate for examining relationships among latent constructs (Hair et al., 2019). The sample size was considered adequate for the PLS-SEM analysis. An *a priori* statistical power analysis based on the most complex endogenous construct, with seven predictors, indicated that a minimum sample size of 103 was required, which was exceeded by the final sample of 290 valid responses (Cohen, 1988; Faul et al., 2009). The analysis proceeded in two stages. First, the measurement model was evaluated in terms of reliability and validity. Second, the structural model was assessed by examining path coefficients and coefficients of determination (*R*^2^). The significance of direct and indirect effects was tested using bootstrapping with 5,000 resamples, and statistical significance was determined based on whether the bias-corrected 95% confidence interval excluded zero (Henseler et al., 2015). Given the cross-sectional nature of the data, the structural paths were interpreted as associations rather than causal effects.

In addition, gender, academic major, English proficiency, and AI-use experience were included as control variables predicting MLBs in the structural model. These control variables were included to examine whether the association between the focal psychological variables and MLBs remained stable after accounting for learner background differences. Because all focal variables were collected through a single self-report questionnaire at one time point, common method bias was also considered (Podsakoff et al., 2003). Procedural remedies included anonymous participation and informing participants that their responses would be used only for academic research purposes. Statistically, common method bias was examined using Harman's single-factor test and full collinearity VIFs (Podsakoff et al., 2003).

## 4 Findings

### 4.1 Descriptive statistics and correlations

Table 4 presents the means, standard deviations, and correlations among the main variables. The mean scores ranged from 3.41 to 3.75, indicating that participants generally reported moderate to moderately high levels of PAII, FE, SE, and MLBs. PAII was positively and significantly correlated with FE (*r* = 0.654, *p* < 0.01), SE (*r* = 0.665, *p* < 0.01), and MLBs (*r* = 0.523, *p* < 0.01). Flow experience was also positively correlated with SE (*r* = 0.614, *p* < 0.01) and MLBs (*r* = 0.535, *p* < 0.01). In addition, self-efficacy was positively correlated with MLBs (*r* = 0.665, *p* < 0.01). Therefore, these correlations were in the expected directions and warranted further testing of the proposed structural model.

**Table 4:** Descriptive statistics and correlations.

Dimensions	Mean	SD	PAII	FE	SE	MLBs
PAII	3.73	0.56	—			
FE	3.41	0.63	0.654^**^	—		
SE	3.60	0.59	0.665^**^	0.614^**^	—	
MLBs	3.75	0.58	0.523^**^	0.535^**^	0.665^**^	—

*N* = 290, ^**^p < 0.01. PAII, perceived AI interactivity; FE, flow experience; SE, self-efficacy; MLBs, motivated learning behaviors.

### 4.2 Structural model evaluation

To assess potential common method bias, Harman's single-factor test was conducted. The results showed that the first unrotated factor accounted for 43.62% of the total variance, which was below the commonly used 50% threshold. This result suggests that common method bias was unlikely to seriously threaten the findings.

The structural model was then evaluated in terms of collinearity, model fit, and explanatory power. As shown in Table 5, all VIF values ranged from 1.51 to 2.92, which were below the recommended threshold of 3.3, indicating that multicollinearity was not a concern. In addition, the SRMR value was 0.067, below the recommended threshold of 0.08, suggesting an acceptable model fit. In terms of explanatory power, the model accounted for 44.9% of the variance in FE (*R*^2^ = 0.449), 50.9% of the variance in SE (*R*^2^ = 0.509), and 50.4% of the variance in MLBs (*R*^2^ = 0.504). Overall, these results suggest that the structural model demonstrated acceptable fit and moderate explanatory power.

**Table 5:** Structural model evaluation.

Criterion	Variable/model	Value	Recommended threshold
VIF	Predictor constructs	1.51–2.92	< 3.3
SRMR	Estimated model	0.067	< 0.08
R^2^	FE	0.449	≥0.25
	SE	0.509	≥0.25
	MLBs	0.504	≥0.25

VIF, variance inflation factor; SRMR, standardized root mean square residual; *R*^2^, coefficient of determination.

### 4.3 Structural model results

The effects of the control variables were also examined. English proficiency was significantly associated with MLBs (β = −0.119, *t* = 2.666, *p* = 0.008), whereas AI-use experience (β = 0.021, *t* = 0.465, *p* = 0.642), gender (β = 0.083, *t* = 0.594, *p* = 0.552), and academic major (β = −0.155, *t* = 1.343, *p* = 0.179) were not significant predictors of MLBs. Importantly, the key hypothesized paths among PAII, flow experience, self-efficacy, and MLBs retained the same pattern of significance after these control variables were included.

The structural model was examined to test the proposed hypotheses. The direct effects are reported in Table 6. As shown in Table 6, PAII was positively associated with FE (β = 0.670, *p* < 0.001) and SE (β = 0.489, *p* < 0.001). In addition, flow experience was positively associated with SE (β = 0.287, *p* < 0.001). With regard to MLBs, self-efficacy was positively associated with MLBs (β = 0.522, *p* < 0.001), whereas the paths from FE to MLBs (β = 0.184, *p* = 0.058) and from PAII to MLBs (β = 0.032, *p* = 0.735) were not significant.

**Table 6:** Structural model results.

Criterion	Predictors	β	*t*	*p*	95% CI
FE	PAII	0.670	16.551	< 0.001	(0.589, 0.746)
SE	PAII	0.489	7.511	< 0.001	(0.360, 0.611)
	FE	0.287	4.160	< 0.001	(0.151, 0.418)
MLBs	PAII	0.032	0.339	0.735	(−0.144, 0.221)
	FE	0.184	1.898	0.058	(−0.019, 0.361)
	SE	0.522	7.595	< 0.001	(0.387, 0.654)
	EP	−0.119	2.666	0.008	(−0.205, −0.031)
	Gender	0.083	0.594	0.552	(−0.191, 0.359)
	Major	−0.155	1.343	0.179	(−0.382, 0.074)
	AI-use experience	0.021	0.465	0.642	(−0.065, 0.115)

PAII, perceived AI interactivity; FE, flow experience; SE, self-efficacy; MLBs, motivated learning behaviors; EP, English proficiency; CI, confidence interval. β values are original sample estimates from SmartPLS. Control variables were included as predictors of MLBs.

The indirect effects were further examined using bootstrapping with 5,000 resamples. As shown in Table 7, the total indirect effect of PAII on MLBs was significant [β = 0.479, *p* < 0.001, 95% CI (0.258, 0.592)]. More specifically, the indirect effect of PAII on MLBs through SE alone was significant [β = 0.255, *p* < 0.001, 95% CI (0.182, 0.341)]. The hypothesized sequential indirect effect of PAII on MLBs through FE and SE was also significant [β = 0.100, *p* = 0.003, 95% CI (0.045, 0.176)]. In contrast, the indirect effect through FE alone was not significant [β = 0.123, *p* = 0.051, 95% CI (−0.014, 0.236)].

**Table 7:** Mediation analysis effects.

Effect	β	*t*	*p*	95% CI
Total effects	0.511	8.578	< 0.001	(0.392, 0.624)
Total indirect effect	0.479	8.114	< 0.001	(0.258, 0.592)
PAII → FE → MLBs	0.123	1.950	0.051	(−0.014, 0.236)
PAII → SE → MLBs	0.255	6.372	< 0.001	(0.182, 0.341)
PAII → FE → SE → MLBs	0.100	2.963	0.003	(0.045, 0.176)

PAII, perceived AI interactivity; FE, flow experience; SE, self-efficacy; MLBs, motivated learning behaviors; CI, confidence interval; β values are original sample estimates from SmartPLS.

**Figure 1:**
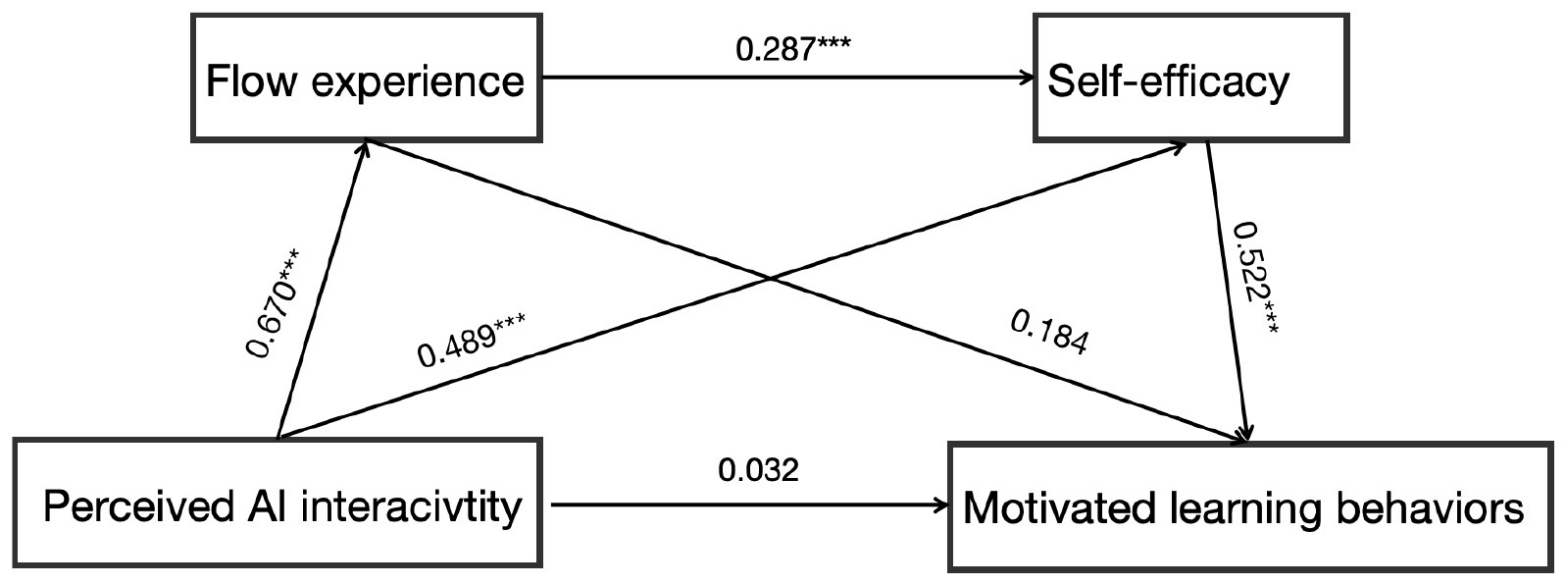
Tested structural model with standardized path coefficients. ***p < 0.001. Paths without significance markers were not statistically significant.

As illustrated in Figure 1, the statistically supported pathways primarily involved SE, either as a sole mediator or as part of the sequential pathway involving FE and SE. By contrast, neither the direct path from PAII to MLBs nor the indirect path through FE alone reached significance. Collectively, these findings suggest that the relationship between PAII and MLBs was mainly explained through pathways involving SE, whereas the simple mediation through FE alone was not supported. Taken together, H1 and H2 were not supported, whereas H3 and H4 were supported.

## 5 Discussion

This study examined how perceived AI interactivity (PAII) was associated with motivated learning behaviors (MLBs) through flow experience and self-efficacy. Overall, the findings suggest that PAII may become behaviorally meaningful mainly when it is connected with learners' psychological processes. From an SCT perspective, this pattern indicates that perceived environmental conditions do not automatically translate into sustained learning behaviors; rather, learners' experiential states and efficacy beliefs play an important role in linking PAII with MLBs.

The non-significant direct association between PAII and MLBs suggests that positive perceptions of AI interactivity may create favorable learning conditions, but may not be sufficient by themselves to promote sustained effort, persistence, and proactive engagement. PAII reflects learners' subjective perceptions of whether AI-supported interaction is responsive, controllable, engaging, and personalized (Wang et al., 2025), whereas MLBs involve more sustained forms of behavioral engagement in learning activities (Gagné and Deci, 2005; Li et al., 2021). This distinction helps explain why the relationship between PAII and MLBs should be understood as psychologically mediated rather than simply direct.

The associations between PAII, flow experience, and self-efficacy further indicate that learners' perceptions of AI interactivity are closely related to their psychological responses during AI-supported learning. When learners perceive AI-supported interaction as responsive, controllable, engaging, and personalized, they may be more likely to experience absorption, enjoyment, and a sense of control, which are central features of flow experience (Csikszentmihalyi et al., 2014; Schmidt et al., 2014; Zhou et al., 2025). At the same time, such perceptions may strengthen self-efficacy by making learning tasks appear more manageable and by providing timely and personalized support. This interpretation is consistent with SCT, which emphasizes the role of personal psychological processes in connecting perceived environmental conditions with behavior (Bandura, 1986).

Self-efficacy appeared to play a more central role than flow experience in explaining MLBs. This may be because flow experience reflects an immediate positive state during task engagement, whereas self-efficacy concerns learners' beliefs that they can complete learning tasks successfully. Such efficacy beliefs are closely related to effort regulation, persistence, and active engagement (Bandura, 1997; Schunk and DiBenedetto, 2020). Therefore, flow experience should not be interpreted as unimportant; rather, its role appears to be more indirect. Positive flow experiences may support MLBs mainly when they help learners develop stronger self-efficacy. In this sense, the sequential pathway from flow experience to self-efficacy provides a more nuanced explanation of how PAII is associated with MLBs in AI-supported EFL learning.

These findings also have theoretical and practical implications. Theoretically, the study suggests that PAII should not be understood only as learners' evaluation of AI tools, but also as a perceived learning condition associated with learners' experiential and cognitive-motivational responses. Practically, the findings suggest that AI designers should not focus only on strengthening learners' perceptions of AI interactivity. Instead, AI-supported learning environments should provide timely feedback, engaging interaction, controllable learning options, and personalized support in ways that help learners experience flow and develop stronger self-efficacy. EFL educators should also provide pedagogical guidance on how students can use AI feedback to complete concrete learning tasks, solve learning problems, and build confidence through successful task completion. Together, these forms of design and instructional support may help positive perceptions of AI interactivity foster flow experience, strengthen self-efficacy, and ultimately support more sustained MLBs.

While these findings provide a more nuanced understanding of the psychological pathways linking PAII to MLBs, several limitations should be acknowledged. Female students and English majors were overrepresented in the sample, accounting for 78.6% and 67.2% of participants, respectively. Although gender and academic major were included as control variables and were not significant predictors of MLBs, the small male subsample and the concentration of English majors may limit the generalizability of the findings to more gender-balanced EFL populations and learners across disciplines. In addition, 55.9% of the participants reported no formal English certification, suggesting that certification status may not fully capture learners' actual English proficiency. Because the subgroup sizes were unequal, subgroup comparisons were not conducted in the present study to avoid unstable estimates. Future research should recruit more balanced samples through stratified or multi-site sampling and use more objective proficiency measures, such as standardized test scores, course grades, or placement test results.

Second, the study relied on a cross-sectional quantitative design. Although this design was suitable for testing the hypothesized model, it could not establish the temporal or causal ordering of the proposed sequential pathway from PAII to flow experience, then to self-efficacy, and finally to MLBs. In particular, although the present study proposed a sequential pathway from PAII to flow experience, then to self-efficacy, and finally to MLBs, the reverse ordering between flow experience and self-efficacy is also plausible, as learners with stronger self-efficacy may be more likely to experience flow in AI-supported EFL learning. Future studies should adopt longitudinal, experimental, or cross-lagged designs to examine alternative and reciprocal pathways among these constructs (Jia et al., 2024; Kohnke et al., 2025).

## 6 Conclusion

This study examined the relationship between PAII and MLBs among Chinese EFL learners, with particular attention to the mediating roles of flow experience and self-efficacy. The findings suggest that PAII is better understood as being associated with MLBs through learners' psychological processes rather than through a simple direct relationship. In particular, self-efficacy appeared to play a central role, both as an independent mediator and as part of the sequential pathway involving flow experience and self-efficacy. Although the indirect path through flow experience alone was not significant, flow experience may still contribute to MLBs when it is connected with stronger self-efficacy beliefs.

The study contributes to research on AI-supported EFL learning by clarifying how PAII is associated with MLBs through flow experience and self-efficacy. It also extends the application of SCT by linking learners' perceptions of AI interactivity within AI-supported learning environments, personal psychological processes, and motivated behavioral outcomes. These findings suggest that learners' positive perceptions of AI interactivity may become more meaningful when they support flow experiences in learning and strengthen learners' beliefs in their ability to complete learning tasks successfully. Overall, the study highlights the importance of supporting both flow experience and self-efficacy in AI-supported EFL learning, so that positive perceptions of AI interactivity can be more effectively connected with sustained MLBs.

## Data Availability

The datasets presented in this study can be found in online repositories. The names of the repository/repositories and accession number(s) can be found in the article/supplementary material.

## Appendix

A survey of the effects of perceived AI interactivity on EFL learners' flow experience, self-efficacy, and motivated learning behaviors

1 AI tools can process my questions quickly.
2 AI tools respond rapidly to my inquiries.
3 I can communicate directly with AI tools at any time to obtain further assistance.
4 I can freely choose the learning content and pace when using AI tools.
5 I can guide AI tools to adjust learning suggestions based on my own learning objectives.
6 I feel highly engaged when learning with AI tools.
7 I can maintain a high level of focus when interacting with AI tools.
8 I am interested in the learning content provided by AI tools and am willing to actively participate.
9 AI tools can meet my specific learning needs.
10 AI tools adjust learning suggestions according to my needs.
11 AI tools continuously optimize learning content based on my learning progress.
12 When learning with AI tools, I feel that time passes very quickly.
13 When interacting with AI tools, I feel happy and joyful.
14 When interacting with AI tools, I feel satisfied because I enjoy the learning process.
15 I feel that the entire process of learning with AI tools is effortless and easy.
16 When interacting with AI tools, I am rarely disturbed by external factors and often forget my surroundings.
17 I believe I can skillfully use AI tools to complete learning tasks.
18 I believe I can use AI tools to find solutions to various learning challenges.
19 I believe I can effectively master new learning content with the help of AI tools.
20 I believe I can effectively integrate AI tool resources to enhance my learning performance.
21 I believe I can remain calm when facing difficulties in AI-supported learning.
22 After learning with AI tools, I am more willing to actively explore related learning resources.
23 After learning with AI tools, I persist in completing tasks even when they are difficult.
24 After learning with AI tools, I proactively take the initiative to solve problems encountered during learning.
25 After learning with AI tools, I demonstrate greater initiative and enthusiasm throughout the learning process.
